# Gut microbial modulation in the treatment of chemotherapy-induced diarrhea with Shenzhu Capsule

**DOI:** 10.1186/s12906-019-2548-y

**Published:** 2019-06-11

**Authors:** Jing Wang, Wuwen Feng, Shiyang Zhang, Lu Chen, Fei Tang, Yongcheng Sheng, Hui Ao, Cheng Peng

**Affiliations:** 1 School of Pharmacy, Chengdu University of Traditional Chinese Medicine, Chengdu, 611137 China; 2 Innovative Institute of Chinese Medicine and Pharmacy, Chengdu University of Traditional Chinese Medicine, Chengdu, 611137 China

**Keywords:** Gut microbiota, Chemotherapy-induced diarrhea, Herbal medicine, 5-fluorouracil

## Abstract

**Background:**

Gut microbiota plays a crucial role in the treatment of gastrointestinal (GI) diseases such as chemotherapy-induced diarrhea (CID). Shenzhu Capsule (SZC) is a Chinese herbal formula, which is composed of Renshen (rhizomes of *Panax ginseng* C. A. Mey.) and Baizhu (rhizomes of *Atractylodes macrocephala* Koidz.). Many Chinese traditional anti-diarrheal formulae that contain Renshen and Baizhu are capable of effectively alleviating CID. However, the efficacy in vivo and potential mechanism of SZC (the form of compatibility of Renshen and Baizhu) in the treatment of CID had not been elucidated. Here, this study aimed to investigate whether SZC exhibited the anti-diarrheal activity, and whether gut microbiota was involved in the therapeutic effect of SZC on CID.

**Methods:**

High performance liquid chromatography (HPLC), gas chromatography-mass spectrometer (GC-MS) and infrared spectroscopy (IR) analyses were used to characterize the extracted components in SZC. The mice were orally administrated with SZC in a preventive mode on the first 2 days of this experiment, and then intraperitoneally injected with 5-FU (40 mg/kg/d) for 6 days. SZC treatment lasted until the 3rd day after the end of 5-FU chemotherapy. We investigated the effects of SZC on body weights, diarrhea, thymus/spleen indexes, colonic tissues, and gut microbiota. Colonic histology was examined by hematoxylin-eosin (HE) staining. 16S rDNA Amplicon Sequencing was used to analyze the gut microbial structure from fecal samples.

**Results:**

SZC significantly increased the body weights and thymus/spleen indexes, alleviated diarrhea, and reversed histopathological changes of colons. In addition, gut microbiota analysis revealed that the overall structure of gut microbiota in CID mice was disturbed, but reversed to the normal state after SZC treatment. At genus level, SZC significantly inhibited the growth of some potential pathogens associated with diarrhea, such as *Clostridiumm*, *Bacteroides*, *Parabacteroides*, *Alloprevotella*, *Acinetobacter* and *Pseudomonas*.

**Conclusions:**

In our study, these data illustrated that SZC inhibited the growth of many potential pathogens during the alleviation of CID. Gut microbial modulation was associated with the anti-diarrheal activity of SZC.

## Background

Gut microbiota, known as a complex ecological community colonized in human gastrointestinal (GI) tract, can interact with host factors to affect normal physiology and diseases [1]. Healthy gut microbiota contributes to the metabolism of nutrition and drugs, structural integrity of intestinal mucosal barrier, immune regulation, and inhibition of pathogens [2, 3]. Gut microbiota in the disordered state is likely to trigger many diseases, such as diarrhea [4], inflammatory bowel disease [5], metabolism disturbances (obesity, insulin resistance and diabetes) [6], and cardiovascular diseases [7]. Due to its ubiquitous roles in the maintenance of gut homeostasis and human health, gut microbiota exhibits great effects on the development and progression of diseases [8, 9].

Among the diseases related to altered gut microbiota, chemotherapy-induced diarrhea (CID) is one of the most common digestive complications in cancer patients treated with chemotherapeutic drugs such as fluoropyrimidines and irinotecan [10]. CID has been found to occur in 50–80% of cancer patients, especially those with advanced cancer [11]. In addition, severe diarrhea (grade 3/4) was reported in 16.6% colorectal cancer patients receiving 5-fluorouracil (5-FU), the first-line chemotherapeutic drug for colorectal cancer [12]. A growing number of evidences showed that CID could directly threaten the lives of patients, or indirectly affect the cycle and efficacy of chemotherapy [13]. Therefore, at present, medication is being introduced into the treatment of CID. Modern medicines such as loperamide and octreotide are often used to relieve CID in clinical practices [14]. However, their therapeutic effects are not satisfactory sometimes. For example, loperamide cannot alleviate several diarrheal features including abdominal cramps, an inability to eat, increasing fatigue, increasing weakness, chest pain, gastrointestinal bleeding [15]. Long-acting octreotide acetate was not effective in preventing or relieving diarrhea in cancer patients undergoing chemoradiation therapy [16]. In a word, there is still a lack of safe and effective modern medicines for the prevention and treatment of CID. Therefore, considerable attentions have been paid to complementary or alternative medicines such as traditional herbal medicines [17, 18].

Shenzhu Capsule (SZC), a formula of Chinese herbal medicine, is composed of ginsenosides and polysaccharides from Renshen (rhizomes of *Panax ginseng* C. A. Mey.), essential oil and polysaccharides from Baizhu (rhizomes of *Atractylodes macrocephala* Koidz.). In the past few years, our researches mainly focused on the therapeutic effects of SZC for gastrointestinal diseases. For example, we had reported that SZC induced apoptosis of gastric cancer cells through mitochondrial pathway [19–21]. In addition, SZC could reversely regulate the expression of some genes (metabolic, ion channels and transport proteins) in SGC-7901 tumor-bearing mice, resulting in the improvement of gastric cancer through multiple targets [22]. Moreover, SZC not only regulated immune and inflammatory responses during the treatment of ulcerative colitis (UC), but also acted on UC complications (bloody diarrhea) and prevented UC from developing into colorectal cancer [23]. At present, our attentions are being paid to investigate whether SZC exhibits therapeutic effect on CID, the GI side effect of chemotherapeutic drugs. As is known, many Chinese traditional anti-diarrheal formulae such as Shenling Baizhu Powder, Buzhong Yiqi Decoction and Sijunzi Decoction, all of which contain Renshen and Baizhu, had been proved to possess the ability of alleviating CID [24–26]. However, no study has determined if SZC (the form of compatibility of Renshen and Baizhu) exerts any therapeutic effect on CID. In recent years, gut microbiota has become an important frontier to reveal the therapeutic mechanisms of medicines [27]. Therefore, it is of great significance to investigate the therapeutic effect of SZC on CID based on gut microbiota.

In this study, we simulated a CID model in mice receiving 5-FU chemotherapy to evaluate the efficacy and interpret the potential mechanism of SZC in the treatment of diarrhea. Both diarrhea rates and histological changes of colonic tissues were recorded. 16S rDNA Amplicon Sequencing was used to analyze the gut microbial structure from fecal samples.

## Methods

### Chemicals and reagents

5-FU injection (25 mg/mL, No. 1708061) was provided by Tianjin JinYao Pharmaceutical Co., Ltd. (Tianjin, China). Standard compounds (purity > 98%) of ginsenosides Rg1, Rc, Rd, Re, Rb1, Rb2 and Rf were purchased from Chengdu Must Bio-Technology Co. Ltd. (Chengdu, China). Other chemicals and reagents were obtained from commercial sources.

### Experimental animals

Twenty-four, 8-week-old SPF graded Kunming mice (12 males and 12 females) (Chengdu Dossy Experimental Animal Co., LTD., Chengdu, China, No. SCXK (chuan) 014–028) were adapted to the laboratory environment for 3 days before the experiment. The mice were housed under the standard environment (temperature of 25 ± 2 °C, humidity of 50 ± 5%, and a 12 h light/12 h dark cycle) with free access to the same water and diets. All efforts were made to minimize animal suffering. All experimental procedures involving animals were agreed by Animal Ethics Committee (Approval No. AEC-201706) in Chengdu University of Traditional Chinese Medicine (CDUTCM) (Chengdu, China).

### Collection of Renshen and Baizhu

The materials were purchased from Lotus Pond Chinese herbal medicine market, Chengdu, Sichuan Province, People’s Republic of China. They were respectively identified as the rhizomes of *Panax ginseng* C. A. Mey. and *Atractylodes macrocephala* Koidz. by Associate Professor Lu Chen from School of Pharmacy in CDUTCM. The voucher specimens of Renshen and Baizhu (No. 17102101 and 17102102, respectively) were deposited at School of Pharmacy in CDUTCM.

### Preparation and characterization of SZC

SZC is composed of ginsenosides and polysaccharides isolated from Renshen, essential oil and polysaccharides isolated from Baizhu. In this study, we extracted and characterized those components according to the preparing process of SZC [24].

### Preparation and high performance liquid chromatography (HPLC) characterization of ginsenosides from Renshen in SZC

Renshen powder (200 g) was refluxed 3 times by 60% ethanol (8 ×) for total 6 h. Then, the combined extracts were concentrated by vacuum-rotary evaporation. The concentrate was eluted by 70% ethanol on D101 macroporous adsorptive resin. All the collected eluents were concentrated, and then dried at 40 °C in a vacuum oven. Final residue of ginsenosides (1.4 mg) was dissolved in 1 mL methanol and then filtered through a 0.45 μm filter membrane. Ginsenosides were characterized by HPLC (1260, Agilent, USA) with a C18 column (250 mm × 4.6 mm i.d., particle size 5 μm) (Cosmosil, Japan). The column was eluted at 35 °C with a detection wavelength at 203 nm and an injection volume of 10 μL. The flow rate of the mobile phase of water (A) and acetonitrile (B) was set at 1.0 mL/min. Gradient separation was based on the following: 0–10 min, 18–21% B; 10–20 min, 21–22% B; 20–30 min, 22–26% B; 30–40 min, 26–30% B; 40–55 min, 30–32% B; 55–75 min, 32–33.8% B; 75–90 min, 33.8–38% B; 90–105 min, 38–45% B; 105–110 min, 45–18% B. And the standard curves were obtained based on the absorbencies of different concentrations of standard ginsenosides Rg1, Re, Rf, Rc, Rd, Rb1 and Rb2.

### Preparation and gas chromatography-mass spectrometer (GC-MS) characterization of essential oil from Baizhu in SZC

Baizhu powder (200 g) was refluxed by distilled water (10 ×) for 12 h. Final essential oil was gathered by steam distillation from the powder. Essential oil was characterized by GC-MS (GC 7890A and MS 5975C, Agilent, USA). Chromatographic separation was achieved on a capillary column HP-5MS (30 m × 0.25 mm i.d.; film thickness 0.25 μm). The electron ionization system operated at 70 eV of ionization energy. Helium was used as the carrier gas. 1 μL sample (essential oil: n-hexane, 1: 100 dilution) was injected and the injector temperature was set at 230.0 °C. Initial oven temperature was set at 100.0 °C, then increased to 120.0 °C at the rate of 3.0 °C/min and held at 120.0 °C for 20 min, and finally increased to 220.0 °C at the rate of 3.0 °C/min. MS was recorded in the range of 35–550 m/z at the rate of 1.0 scan/s. Identification of different components in essential oil was based on the comparison with the known constituents that stored in National Institute of Standards and Technology Library (NIST14.0).

### Preparation and infrared spectroscopy (IR) characterization of polysaccharides from Renshen and Baizhu in SZC

Renshen (200 g) or Baizhu (200 g) powder was refluxed by distilled water (10 ×) for 2 h. The procedure was repeated 3 times. Then these extracted solutions were combined, concentrated, and cooled (4 °C) for 12 h. The cooled concentrate solution was precipitated in 60% ethanol, and final residues were dried at 40 °C in a vacuum oven. The characterization was carried out on a FTIR Spectrum (cary 660, Agilent, USA). Polysaccharides (1 mg) from Renshen or Baizhu were mixed with 150 mg KBr and homogenized by a pestle in the mortar. The mixture was then made by a hydraulic press into a slice, which was analyzed in IR at the wavenumber region from 400 to 4000 cm^− 1^.

### Experimental design

According to the clinical dosages of Renshen (3 g per time) and Baizhu (3 g per time) (two Chinese medicines in SZC) in China Phannacopeia [22, 28, 29], we chose the oral doses of ginsenosides and polysaccharides from Renshen as 126.7 mg/kg/d and 176.3 mg/kg/d respectively, and the oral doses of essential oil and polysaccharides from Baizhu as 35.3 μL/kg/d and 192.3 mg/kg/d respectively for mice. SZC was mixed in 0.1% sodium carboxymethyl cellulose solution. Twenty-four mice were randomly assigned into three groups (*n* = 8): the control, model and SZC groups, with 4 males and 4 females per group. Our modeling method was based on the existing literature [30]. The mice were orally administrated with SZC in a preventive mode on the first 2 days of the experiment, and then intraperitoneally injected with 5-FU (40 mg/kg/d) for 6 days. Mice in the SZC group were orally administrated with SZC half an hour before 5-FU chemotherapy. SZC treatment lasted until the 3rd day after the end of 5-FU chemotherapy. Mice in the control group received equal volume of liquid solvent for the whole 11 days, while the untreated mice in the model group received 5-FU chemotherapy on the 3–8 days.

Both body weights and diarrhea states were recorded every day. For the diarrhea evaluation, the mice with moist and unformed stools, or perianal stains, were deemed to show the clinical symptoms of diarrhea. The calculation formula of diarrhea rates in each group was given as follow: the diarrhea rate (%) = the number of diarrheal mice/the number of mice × 100%. Twenty-four hours after the last administration, fecal sample (4 g) was collected in sterile plastic tube and stored at − 80 °C. All the animals were sacrificed by cervical dislocation. Then thymuses and spleens were removed after the sacrifice of mice. Thymus and spleen indexes of each mouse were calculated according to the following formula: thymus (spleen) index = thymus (spleen) weight/body weight (mg/g).

### Histological observation

The colon segments (2 cm) of each mouse were collected and fixed in 4% formaldehyde solution after the sacrifice of mice. Then the colonic samples were embedded by paraffin, sliced (4 μm), and stained with hematoxylin-eosin (HE). The histological changes of colonic segments were observed with the microscope (DMI300B, Leica, Germany).

### Gut microbiota analysis

The genomic DNA of fecal samples was extracted by sodium dodecyl sulfate (SDS). After that, the concentration of DNA was detected using agarose gel electrophoresis. The V3–V4 region of bacterial 16S rRNA gene was amplified by polymerase chain reaction (PCR) with 341F–806R primer set. All PCR reactions were induced by High-Fidelity PCR Master Mix (New England Biolabs, USA). PCR products were detected by electrophoresis with 2% agarose gel, and then purified by Qiagen Gel Extraction Kit (Qiagen, Germany). TruSeq® DNA PCR-Free Sample Preparation Kit (Illumina, USA) was used to generate sequencing library. Library quality was evaluated on Qubit@2.0 Fluorometer (Thermo Scientific, USA) and Agilent Bioanalyzer 2100 system. Finally, the Library was conducted on Illumina HiSeq 2500 platform (Novogene, Beijing, China).

### Bioinformatics analysis

Paired-end reads of each sample were merged by FLASH (Version 1.2.7) to get raw tags. High-quality clean tags were generated from the strict filtration of raw tags by QIIME (Version 1.9.1). Final effective tags were produced after the removal of chimera sequences in the tags, which were detected by gold database (Version 1.9.1) and UCHIME Algorithm. The sequences at 97% identity were clustered into the same operational taxonomic units (OTUs) by Uparse (Version 7.0.1001). Then the representative sequence of each OTU was selected for the species annotation. MUSCLE (Version 3.8.31) was used for the multiple sequence alignments to get the phylogenetic relationships. The OTUs abundance information was normalized by the sequence number standard. ACE and Shannon indexes that respectively indicated richness and diversity of gut microbiota were calculated on QIIME (Version 1.7.0). Linear discriminant analysis effect size (LEfSe) was used to detect the dominant bacterial community difference among groups. The lowest screening value of linear discriminant analysis (LDA) in LEfSe software was defaulted to 4. Venn, LEfSe and Non-Metric Multi-Dimensional Scaling (NMDS) diagrams were built on R software (Version 2.15.3). Unweighted Pair-group Method with Arithmetic Means (UPGMA) Clustering was performed on QIIME software (Version 1.7.0).

### Statistical analysis

Statistical analysis was performed on SPSS (Version 21). Data were presented as means ± Standard Error of Mean (SEM). One-way analysis of variance (ANOVA) followed by Least Significant Difference (LSD) test was used to analyze the statistical differences between multiple groups. Kruskal-Wallis test was applied if the data did not meet the assumptions of ANOVA. A value of *P* < 0.05 was considered statistically significant.

## Results

### Chemical compositions of SZC

**Fig. 1:**
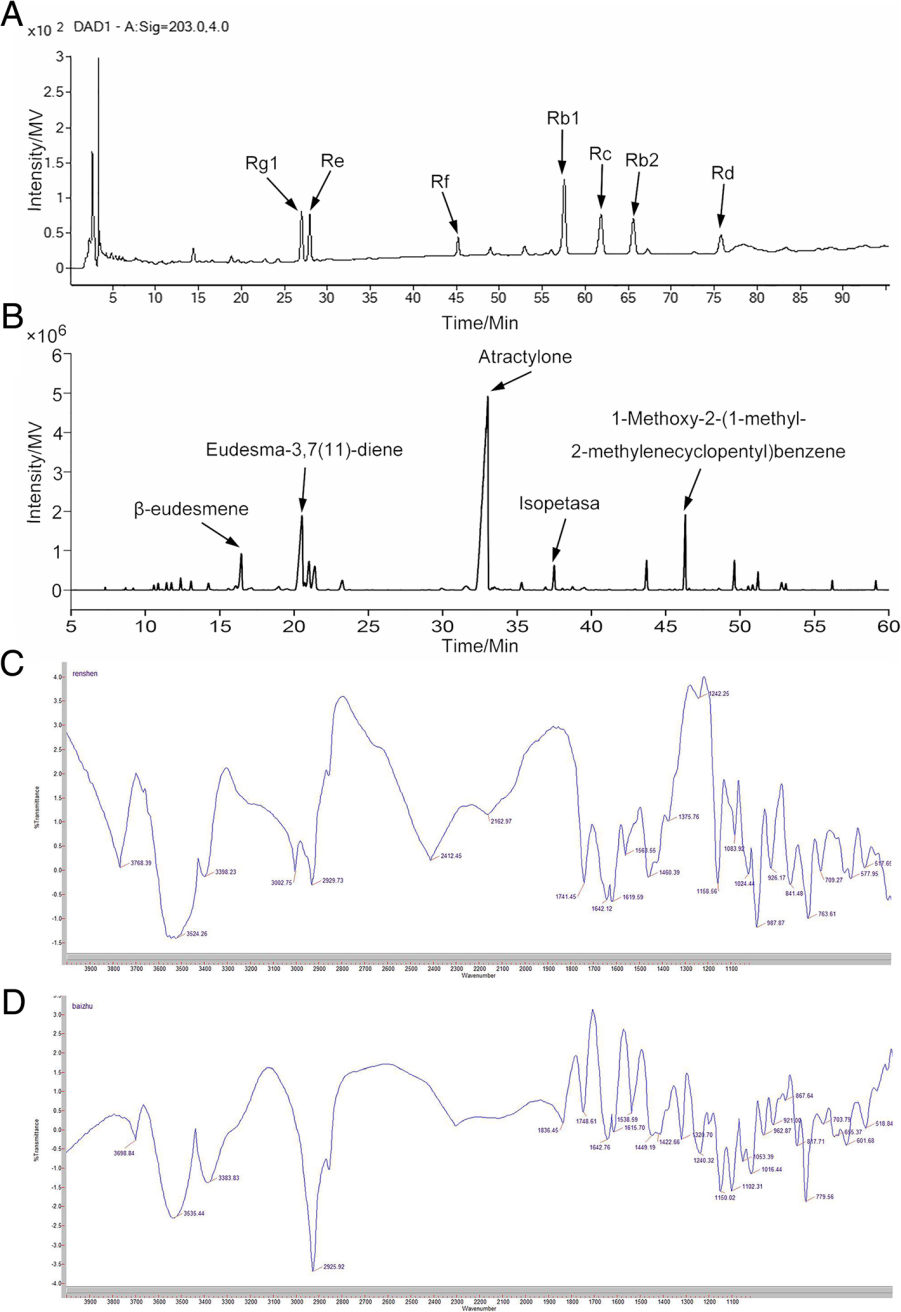
Chemical characterization in SZC. (**a**) HPLC profile of ginsenosides from Renshen. (**b**) GC-MS profile of essential oil from Baizhu. (**c**) IR absorption spectrum of polysaccharides from Renshen. (**d**) IR absorption spectrum of polysaccharides from Baizhu

As shown in Table 1, the quantitations of ginsenosides standards showed good linearities within 6.25–200.00 μg/mL. The compositions of ginsenosides Rg1, Re, Rf, Rb1, Rc, Rb2 and Rd in total ginsenosides were 46.98 ± 0.25, 55.25 ± 0.66, 12.81 ± 0.11, 132.81 ± 0.96, 83.86 ± 0.39, 70.66 ± 0.34, and 31.61 ± 0.32 mg/g, respectively (Fig. 1a). As shown in Fig. 1b, the five main constituents such as *β*-eudesmene (3.82%), eudesma-3,7(11)-diene (12.49%), atractylone (51.67%), isopetasan (2.07%), and 1-methoxy-2-(1-methyl-2-methylenecyclopentyl) benzene (5.10%) were identified in essential oil of Baizhu. Atractylone was considered as the representative constituent of essential oil in Baizhu.

**Table 1:** Linear relationships with correlation coefficients for ginsenosides standards

Standards	Linear relationships	*r*	Linear range(μg/mL)
Rg1	*Y* = 18.72 *X* + 40.44	0.9999	6.25–200.00
Re	*Y* = 14.48 *X* + 40.82	0.9995	6.25–200.00
Rf	*Y* = 20.58 *X* + 55.34	0.9993	6.25–200.00
Rb1	*Y* = 13.99 *X* + 47.49	0.9995	6.25–200.00
Rc	*Y* = 14.30 *X* + 25.17	0.9998	6.25–200.00
Rb2	*Y* = 13.79 *X* + 13.62	0.9999	6.25–200.00
Rd	*Y* = 16.78 *X* + 25.37	0.9995	6.25–200.00

IR scanning results showed that both polysaccharides extracts of Renshen and Baizhu showed characteristic functional groups of polysaccharides (Fig. 1c and d). The bands at 3524.26 cm^− 1^ and 3535.44 cm^− 1^ were due to O-H groups of polysaccharides from Renshen and Baizhu, respectively. The C=O groups in Renshen and Baizhu polysaccharides caused the increases of the absorption bands at 1741.45 cm^− 1^ and 1748.61 cm^− 1^, respectively. In addition, the bands at 1158.56 cm^− 1^ and 1150.02 cm^− 1^ (polysaccharides of Renshen and Baizhu, respectively), which were also the characteristic absorption bands of polysaccharides, resulted from the stretching vibration of C-O in C-O-C cyclic ether.

### SZC ameliorates diarrhea and increases thymus/spleen indexes in mice with CID

**Fig. 2:**
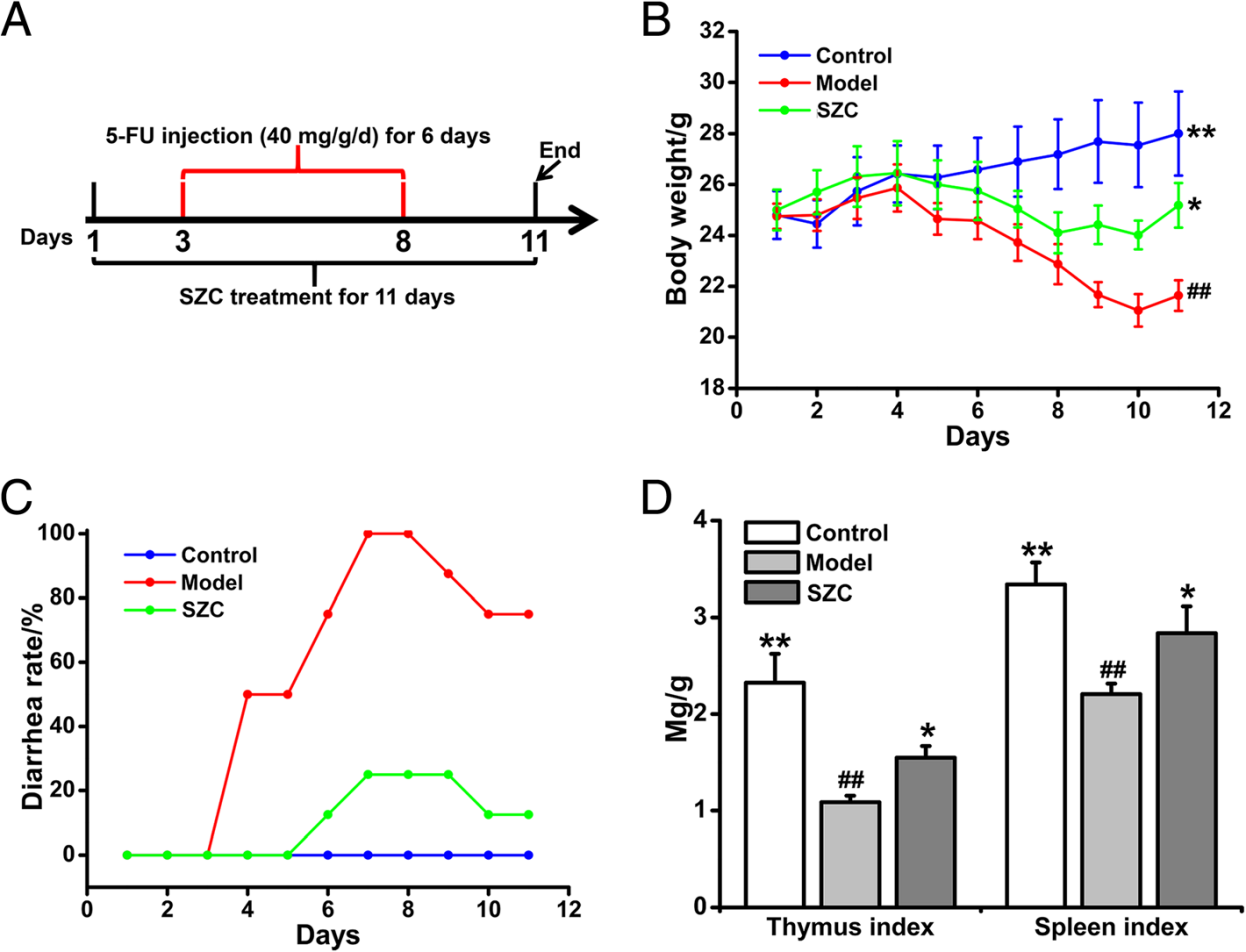
The effects of SZC on body weight, diarrhea, and thymus/spleen indexes in mice with CID (*n* = 8, male/femal = 4). (**a**) Experimental process of CID. (**b**) The daily-changed body weight of each group. (**c**) The daily-changed diarrhea rate of each group. (**d**) Thymus and spleen indexes of each group. Data were presented as means ± SEM. ^*^P < 0.05 and ^**^P < 0.01 compared with model, ^#^*P* < 0.05 and ^##^*P* < 0.01 compared with control

The mice were injected with 5-FU for 6 days during the treatment of SZC (Fig. 2a). During the first 4 days, there was no significant difference in body weights among the control, model and SZC groups. Mice in the model group injected with 5-FU experienced weight loss compared with the control group from days 5–10, and had the lowest weight at the end of the experiment. Treatment with SZC significantly prevented weight loss in CID mice (Fig. 2b). From days 4–8, diarrhea rates increased in the untreated model group, while the clinical symptoms of diarrhea were not observed in the control group. At all time points, the diarrhea rates of the SZC group were obviously lower than those of the model group (Fig. 2c). In addition, the thymus and spleen indexes in the model group decreased significantly after 5-FU chemotherapy, whereas SZC markedly inhibited the decreases of thymus and spleen indexes (Fig. 2d).

### SZC reduces colonic pathological changes in mice with CID

**Fig. 3:**
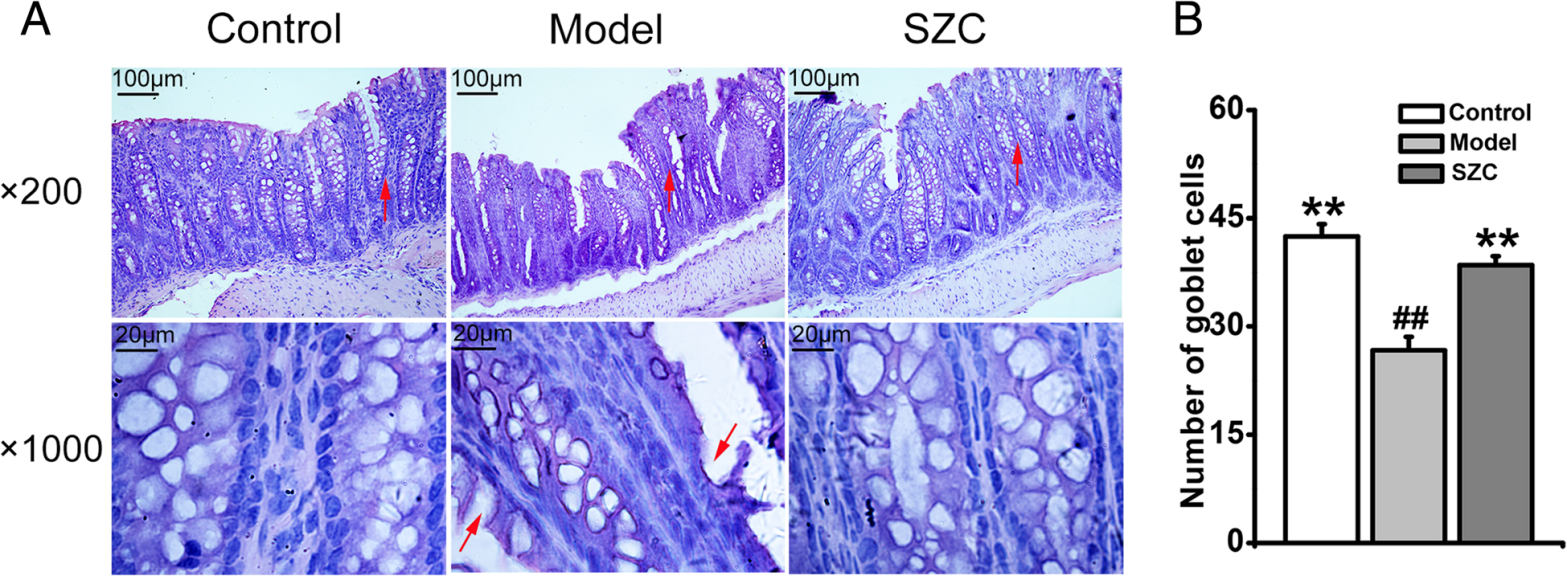
The effects of SZC on histological changes of colon sections in mice with CID (n = 8, male/femal = 4). (**a**) Representative images of HE-stained colonic sections (200 × and 1000 × magnifications). (**b**) Number of goblet cells in HE-stained colonic sections (1000 × magnification). Red arrows in colonic sections (200 × magnification) indicated amplified target areas of pictures, and red arrows in colonic sections (1000 × magnification) indicated damaged goblet cells. Data were presented as means ± SEM. ^*^P < 0.05 and ^**^P < 0.01 compared with model, ^#^*P* < 0.05 and ^##^*P* < 0.01 compared with control

HE-stained colonic section of the model group exhibited remarkable destructions of colonic mucosa and surface epithelia, which were not observed in the control group. Also, in the model group, goblet cells were obviously damaged by 5-FU, and the numbers of goblet cells decreased significantly compared with the control group. However, these histological changes in colonic section were significantly reduced by SZC (Fig. 3).

### SZC modulates the overall structure of gut microbiota in mice with CID

**Fig. 4:**
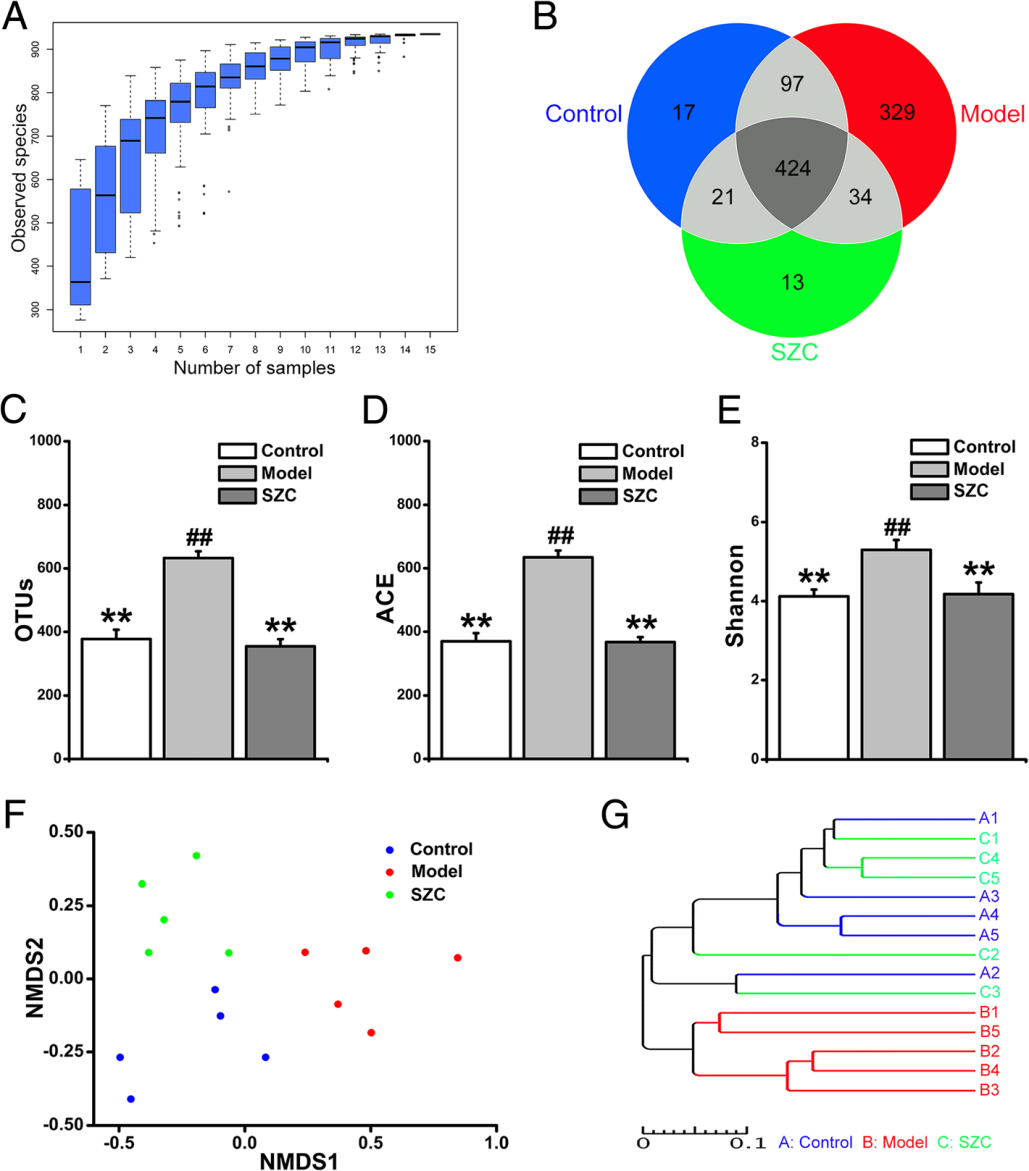
The effects of SZC on the overall structure of gut microbiota in mice with CID (*n* = 5, male/femal = 2–3). (**a**) Species accumulation boxplot was used for the estimations of samples number and species richness. (**b**) Venn diagram indicated the overlap of OTUs in the feces. (**c**) OTUs were clustered from high-quality sequences with 97% similarity. (**d**–**e**) ACE and Shannon values indicated the bacterial community richness and diversity, respectively. (**f**) NMDS plot showed distinct structural changes of the overall bacterial community in each group. (**g**) UPGMA clustering was used to interpret the distance matrix of each sample by the average linkage. Data were presented as means ± SEM. ^*^P < 0.05 and ^**^P < 0.01 compared with model, ^#^*P* < 0.05 and ^##^*P* < 0.01 compared with control

To detect structural differences in gut microbiota between the three groups, a total of 883,537 sequences and an average of 58,902 sequences per group were obtained from the 15 samples. Figure 4a showed that the position of box diagram in species accumulation boxplot tended to be gentle as the increase of samples number, which suggested that samples number and species richness both were adequate and stable. At this sequencing depth, the overall bacterial diversity had already been covered. Then the sequences were clustered into OTUs at 97% similarity, and 935 OTUs in total were obtained from 15 fecal samples. As shown in Fig. 4b, there were 424 OTUs in all three groups, and 17, 329 and 13 unique OTUs were detected in the control, model and SZC groups, respectively. Additionally, the model group showed a significant increase in the number of OTUs compared with the control group. But no significant difference of OTUs number existed between the control and SZC groups (Fig. 4c). Consistent with the changes of OTUs, both ACE richness and Shannon diversity values of bacterial community were significantly increased in the model group. After the treatment of SZC, ACE richness and Shannon diversity values reduced to the normal levels (Fig. 4d and e). NMDS plot showed that the structure of overall bacterial community in the model group was quite different from those in the control and SZC groups (Fig. 4f). The clustering tree based on UPGMA also showed a significant separation in gut microbiota between the control and model groups. Notably, gut microbiota of mice treated with SZC did not significantly differ from that of the control group (Fig. 4g).

### SZC modulates the key phylotypes of gut microbiota in mice with CID

**Fig. 5:**
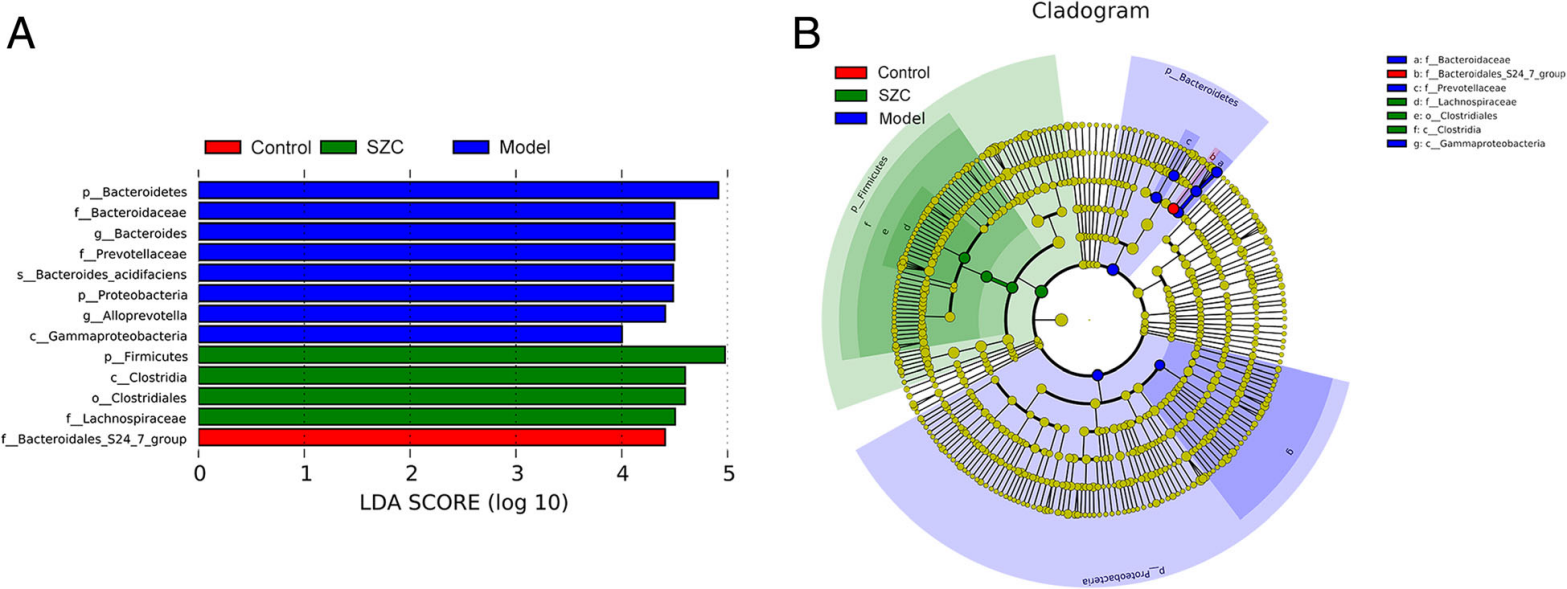
Statistical difference in dominant microbiota among groups (n = 5, male/femal = 2–3). (**a**) The histogram of LDA value distribution. (**b**) The evolutionary branch graph

The histogram of LDA value distribution showed that there were significant differences in dominant microbiota among the three groups (Fig. 5a). 1, 8 and 4 taxa of dominant communities were found in the control, model and SZC groups, respectively. *Bacteroidales_S24-7_group* was the dominant bacteria in the control group. The gut microbiota in the model group was dominated by *Bacteroidetes*, *Bacteroidaceae*, *Prevotellaceae*, *Bacteroides* and *Alloprevotella*. Dominant microbiota in the SZC group included *Firmicutes* and *Clostridia*. An evolutionary branch graph based on LDA scores further revealed the important microbiota in the three groups. The results showed that *Bacteroidales_S24-7_group* was identified as the dominant bacteria in the control group. In the model group, predominant microbiota consisted of *Bacteroidaceae* and *Prevotellaceae*. *Lachnospiraceae*, *Clostridiales* and *Clostridia* were the predominant flora in the SZC group (Fig. 5b).

**Fig. 6:**
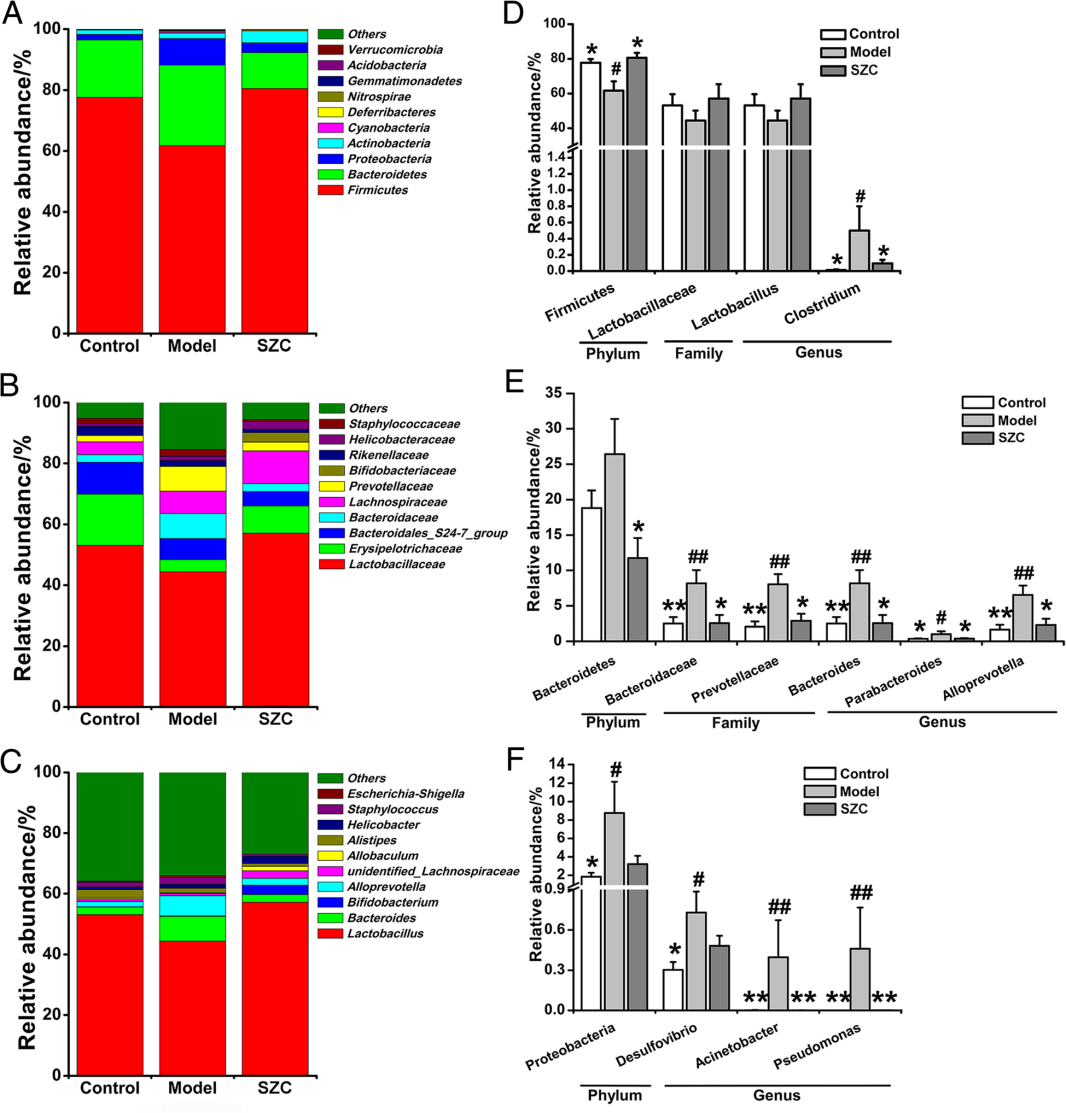
The effects of SZC on the key phylotypes of gut microbiota in mice with CID (n = 5, male/femal = 2–3). (**a**) Relative abundance of different bacterial phyla. (**b**) Relative abundance of different bacterial families. (**c**) Relative abundance of different bacterial genera. (**d**) Relative abundances within *Firmicutes*. (**e**) Relative abundances within *Bacteroidetes*. (**f**) Relative abundances within *Proteobacteria*. Data were presented as means ± SEM. ^*^P < 0.05 and ^**^P < 0.01 compared with model, ^#^*P* < 0.05 and ^##^*P* < 0.01 compared with control

Histograms with respect to phylum, family and genus were used to reveal the microbial species and their relative abundances (Fig. 6a–c). In fecal samples, *Firmicutes*, *Bacteroidetes* and *Proteobacteria* were the 3 dominate phyla, whose relative abundances were 77.62, 18.83 and 1.83% respectively in the control group. The abundances of *Bacteroidetes* (26.41%) and *Proteobacteria* (8.76%) increased in the model group, whereas the abundance of *Firmicutes* (61.75%) decreased. However, SZC treatment could restore the proportions of *Firmicutes* (80.54%), *Bacteroidetes* (11.74%) and *Proteobacteria* (3.22%) after 5-FU chemotherapy. At family level, our result showed that relative abundances of families such as *Bacteroidaceae* (8.19%) and *Prevotellaceae* (8.03%) significantly increased in the model group, compared with those (2.49 and 2.04%, respectively) in the control group. Moreover, SZC treatment significantly reduced the abundances of *Bacteroidaceae* (2.53%) and *Prevotellaceae* (2.87%).

In addition, at genus level, the model group gave rise to high abundances of some potential pathogens such as *Clostridiumm* (0.50%), *Bacteroides* (8.19%), *Parabacteroides* (1.01%), *Alloprevotella* (6.53%), *Desulfovibrio* (0.73%), *Acinetobacter* (0.40%) and *Pseudomonas* (0.46%). However, the relative abundances of these genera in the control group were significantly different, with *Clostridiumm*, *Bacteroides*, *Parabacteroides*, *Alloprevotella*, *Desulfovibrio*, *Acinetobacter* and *Pseudomonas* constituting 0.01, 2.49, 0.35, 1.66, 0.30, 0.00% and 0, respectively. In the SZC group, the inhibitory effects on *Clostridiumm* (0.10%), *Bacteroides* (2.53%), *Parabacteroides* (0.37%), *Alloprevotella* (2.28%), *Desulfovibrio* (0.48%), *Acinetobacter* (0.00%) and *Pseudomonas* (0.00%) were observed compared with those in the untreated model group (Fig. 6d–f).

## Discussion

In this study, all the model mice showed the clinical symptoms of diarrhea. We also found that thymus and spleen indexes in the model mice decreased significantly compared with those of the control group. Thymus and spleen indexes, whose levels depend on the extent of lymphocyte proliferation, can be used to partly reflect host immune function [31]. The results indicated that the immune system of the model group was affected by 5-Fu chemotherapy. In addition, some histological changes of colonic sections were observed in model mice. Especially, the numbers of goblet cells in the model group were remarkably lower than those of the control group. Goblet cells play important roles in producing and maintaining protective mucus blankets by synthesization and secretion of high molecular weight glycoprotein such as mucin. The reduction of goblet cell number could directly or indirectly impact intestinal health [32]. However, diarrhea and intestinal histological changes (Fig. 2) were significantly alleviated by SZC, which indicated that SZC was effective in the treatment of CID. More importantly, the potential mechanism behind this anti-diarrheal effect of SZC needed to be investigated in this study.

Previous studies have demonstrated that the pharmacological effects of Renshen and Baizhu were closely associated with gut microbiota. Renshen could effectively reduce body weight and change gut microbial composition in obese middle-aged Korean women [33]. And Baizhu was capable of reducing intestinal pH value, improving the adhesion of *Bacillusbifidus*, and fostering the normal gut microbiota in lincomycin-induced mice [34]. Therefore, SZC, a formula composed of active herbal extracts from Renshen and Baizhu, might exert potentially modulatory effects on gut microbiota. In addition, gut microbiota is a contributing factor of many diseases including diarrhea [35]. Therefore, we further analyzed gut microbiota from the feces of CID mice.

Gut microbial composition in fecal samples was determined by 16S rDNA Amplicon Sequencing. Species accumulation boxplot indicated that the species richness of gut microbiota was sufficient and stable for the following analyses when the total number of samples was 15 (*n* = 5 in each group) (Fig. 4a). As shown in Fig. 4d and e, gut microbial richness and diversity were significantly increased in the model group. In general, the richness and diversity of microbial populations should be reduced under a pathological condition. The possible cause behind our results was that the richness and diversity of original microbiota was not reduced, and some potential pathogenic bacteria was introduced or increased in CID mice. Our results also showed that microbial richness and diversity decreased to normal levels after SZC treatment. Furthermore, the remarkable differences of distance existed among the three groups in NMDS plot, but a shorter distance was presented between the control and SZC groups. UPGMA clustering showed no obvious structural shift between the SZC and control groups, whereas the gut microbiota in the model group significantly deviated from that of the control group. Taken together, these results demonstrated the overall structure of gut microbiota in CID mice could be reversed to normal by SZC treatment.

Redundancy information uncovered the structural changes in the key phylotypes of gut microbiota based on the following classification: phylum, family and genus. Statistical results revealed that the abundances of *Firmicutes* and *Proteobacteria* significantly changed in the model group. But there was no difference between the SZC and control groups in the abundances of *Firmicutes* and *Proteobacteria*. Also, mice in the model group significantly increased the abundances of some genera such as *Clostridiumm*, *Bacteroides*, *Parabacteroides*, *Alloprevotella*, *Desulfovibrio*, *Acinetobacter* and *Pseudomonas*, which might provide opportunities for the occurrence of diarrhea. For example, high-abundant *clostridium* belonging to *Firmicutes* is one of the causes of diarrhea [36]. *Bacteroides* (*Bacteroidaceae* family and *Bacteroidetes* phylum), a complex organism colonized in the gut, may be the key factor influencing the integrity and immune development of intestinal mucosa in the process of diarrhea. Increased *Bacteroides* may also induce inflammation or even carcinogenesis [37, 38]. *Parabacteroides* in high abundance was found in immune-related diarrhea [39]. In addition, *Alloprevotella* (*Prevotellaceae* family) is an anaerobic Gram-negative bacilli with the high relative abundance in patients with inflammatory bowel disease (IBD) [40]. Both *Desulfovibrio* and *Acinetobacter* (*Proteobacteria* family) were associated with bloody diarrhea [41, 42]. *Pseudomonas* is commonly found in patients with malnourished and infectious diarrhea [43, 44]. These above studies indicated that the growth of these genera (potential pathogens) was associated with diarrhea. As shown in Fig. 6, SZC treatment reduced the abundances of these genera in CID mice. Therefore, our results suggested that SZC could inhibit the growth of potential pathogens and modulated microbial structure in the feces. Moreover, *Lactobacillus* is an important genus for relieving diarrhea [45]. On the contrary, no significant difference in the abundances of *Lactobacillus* (probiotic) existed among the three groups, which was not in accordance with the results of 5-FU-induced decrease of *Lactobacillus* in the previous study [46]. The reason behind the difference might be that our results were based on fecal microbiota in mice, while the previous study focused on fecal microbiota in rats. Therefore, the relationship between *Lactobacillus* and CID still needs a deeper investigation. Conclusively, these results suggested that amelioration of CID with SZC treatment might be mainly mediated by inhibition of potential pathogens.

Although SZC could modulate gut microbial structure of fecal samples in CID mice, the specific relationship between the anti-diarrheal activity of SZC and gut microbiota remained undefined. For example, fecal collection in a non-invasive manner was chosen as the sampling method for gut microbial analysis. However, fecal communities may not completely represent bacterial communities living in intestines, although they play an important role in maintaining intestinal homeostasis and microbial diversity [47, 48]. In addition to structural modulation of gut microbiota, the therapeutic effects of herbal drugs could also be influenced by gut microbial metabolism and pharmacokinetics [49]. Therefore, there is still no systematic and comprehensive interpretation for the association between the anti-diarrheal effect of SZC and gut microbiota, which will be our focus in the future researches.

## Conclusion

In conclusion, our study found that gut microbial structure was modulated by SZC (the Chinese herbal formula) during the alleviation of CID. Especially, at genus level, SZC significantly inhibited the growth of potential pathogens closely related to diarrhea, including *Clostridiumm*, *Bacteroides*, *Parabacteroides*, *Alloprevotella*, *Acinetobacter* and *Pseudomonas*. These results indicated that gut microbial modulation was associated with the anti-diarrheal activity of SZC.

## Data Availability

The data supporting this study are available from the corresponding author upon reasonable request.

## Abbreviations

5-FU: 5-Fluorouracil
ANOVA: Analysis of variance
CDUTCM: Chengdu University of Traditional Chinese Medicine
CID: Chemotherapy-induced diarrhea
GC-MS: Gas chromatography-mass spectrometer
GI: Gastrointestinal
HE: Hematoxylin-eosin
HPLC: High performance liquid chromatography
IBD: Inflammatory bowel disease
IR: Infrared spectroscopy
LDA: Linear discriminant analysis
LEfSe: Linear discriminant analysis effect size
LSD: Least Significant Difference
NMDS: Non-Metric Multi-Dimensional Scaling
OTUs: Operational taxonomic units
PCR: Polymerase chain reaction
SDS: Sodium dodecyl sulfate
SEM: Standard Error of Mean
SZC: Shenzhu Capsule
UC: Ulcerative colitis
UPGMA: Unweighted Pair-group Method with Arithmetic Means
